# Editorial: Lymph Node T Cell Dynamics and Novel Strategies for HIV Cure

**DOI:** 10.3389/fimmu.2018.02950

**Published:** 2018-12-12

**Authors:** Constantinos Petrovas, Vijayakumar Velu

**Affiliations:** ^1^Tissue Analysis Core, Immunology Laboratory, Vaccine Research Center, NIAID, NIH, Bethesda, MD, United States; ^2^Emory Vaccine Center, Yerkes National Primate Research Center, Emory University, Atlanta, GA, United States

**Keywords:** HIV, lymph node, cure, T cells, follicular T helper cells

Currently, more than 36 million people are infected with HIV. Although the introduction of highly active anti-retroviral therapy (HAART) (1, 2) has led to substantial advances in the clinical management of HIV infected individuals, HAART cannot completely eliminate the virus (3). This is because CD4 T helper cells, harboring the virus, remain dormant reservoirs. These reservoirs are difficult to measure and are present even in HAART-treated HIV infected individuals with undetectable levels of HIV in the blood. A growing body of studies has revealed follicular helper (Tfh) CD4 T cells, a highly differentiated CD4 T cell population localized in immunologically sanctuary sites (follicle/germinal center) (4), as a major reservoir of HIV (5). The present Frontiers in Immunology eBook compiles 16 timely review articles focusing on the dynamics of major follicular immune cell types in HIV/SIV infection and their potential role for disease pathogenesis and the viral persistence in the lymph node (Figure 1).

**Figure 1:**
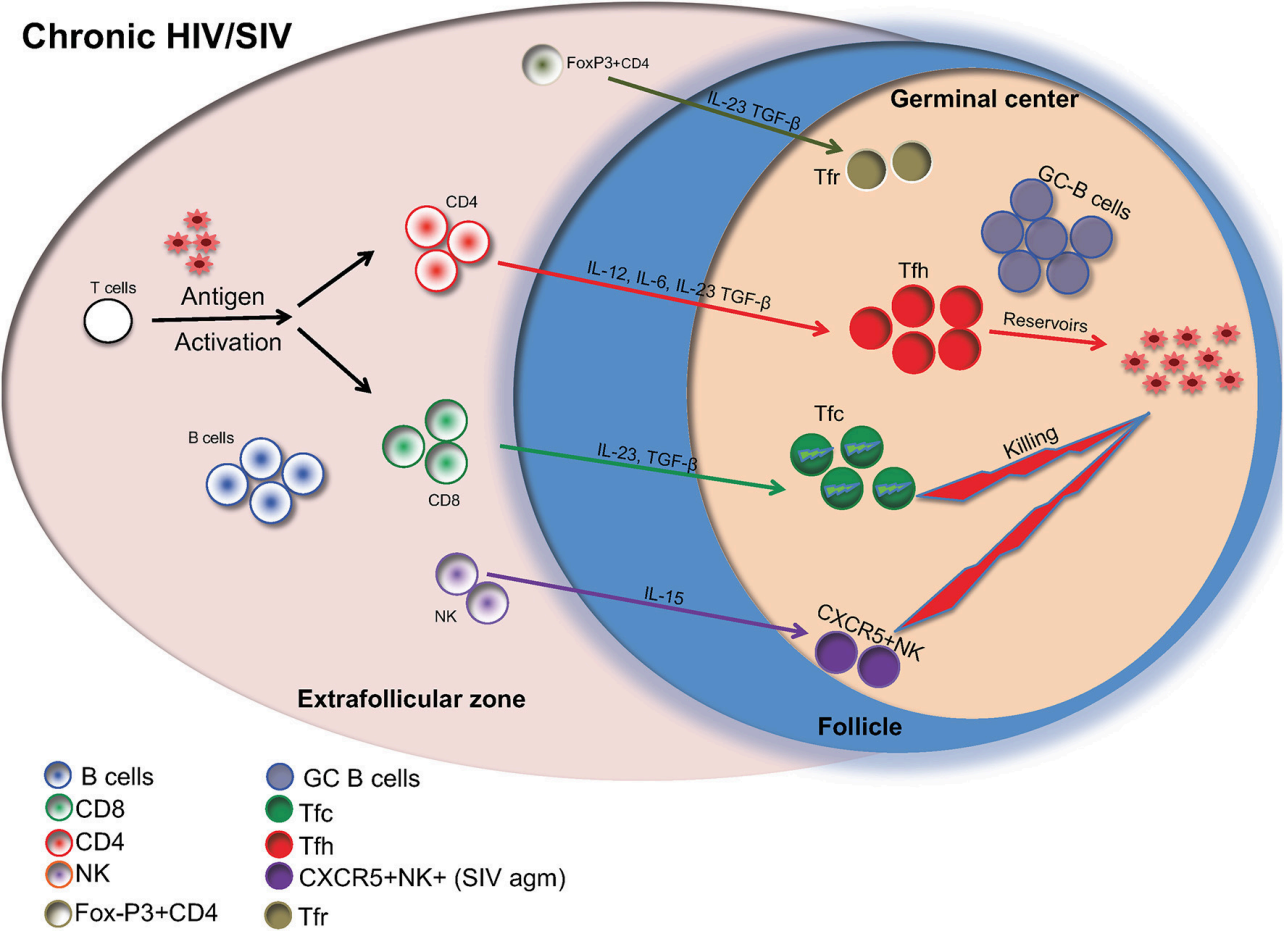
Major follicular cell populations in chronic HIV/SIV infection are shown. Cytokines are important for the differentiation of relevant cells as well as their possible contribution to reservoir formation is also shown.

Vaccari and Franchini provide an overview of follicular T cell populations in the SIV NHP model. The phenotype and function of NHP Tfh cells as well as their role in viral reservoir formation and the impact of HIV/SIV infection on Tfh dynamics is discussed. Besides their role as immune suppressors, the possible role of Tfr cells in the development of high avidity antigen-specific antibodies is discussed. The recent literature for follicular CD8 T cell dynamics and *in vivo* manipulation to study their role is reviewed. The authors, convincingly reveal the importance of NHP studies to understand the follicular dynamics in HIV/SIV pathogenesis and explore *in vivo* manipulations targeting these cell populations. Investigation of follicular CD4 T cell heterogeneity is an important parameter for the understanding of generation of neutralizing antibodies as well as the formation of viral reservoir. Velu et al. discuss several follicular CD4 T cell subsets including CXCR3^+^ Th1-like follicular helper CD4 T cells. High levels of IFNg and IP-10 observed in HIV/SIV could represent a mechanism for the differentiation of Tfh toward a Tfh1 phenotype. Given the differential levels of CCR5 expression by Tfh subsets, future studies should take under consideration this heterogeneity when the virus reservoir is under investigation. Tfh1 cells were found in vaccinated non-human primates the authors suggest that local inflammatory signals could represent critical regulators of Tfh1 cell dynamics. The authors show the need for further mechanistic studies aiming to understand the dynamics of follicular CD4 and CD8 T cells. Tracking the movement of cells between different anatomical compartments in human subjects is highly challenging. Analysis of phenotypically alike cells could represent a starting point for the investigation of cell subsets from compartments with possible dynamic interchange. Banga et al. provide data showing that circulating CXCR3^+^CXCR5^−^ CD4 T cells is the major blood compartment containing replication competent virus in cART, aviremic individuals. Among the parameters analyzed, the frequency of PD-1^+^ cells was significantly correlated with the enrichment of replication competent virus in the circulating CXCR3^+^ CD4 T cell compartment. The data suggest a connection between the presence of infected lymph node and circulating CXCR3+ CD4 T cells. The potential role of CD8 T cells in viral control has been shown by several HIV/SIV studies. Xiao et al. review recent data on CD8 T cells expressing the receptor CXCR5 (fCD8 T cells) and have the ability to migrate into follicular areas. A comparison between fCD8 dynamics in chronic LCMV and HIV infection revealed key transcriptional regulation of these cells in the setting of chronic viral infections. However, what regulates their intra-lymph node trafficking is still unknown. The authors comment on the capacity of the fCD8 T cells for cytokine production and ability for suppressing chronic viral infection. Given this profile, strategies for therapeutic use of fCD8 to purge the virus are also discussed. The dynamics of Tfh CD4 T cells is the outcome of a complex process regulated by multiple factors including tissue inflammation, antigenic stimulation and local immunosuppressor mechanisms. Jaio et al. provide original data regarding the functionality of HIV-specific CXCR5^+^CD8^+^ T cells in the peripheral blood as well as the role of PD-1 as regulator of this functionality. Furthermore, distribution of CCXR5+CD8 T cells in the lymph node negatively correlated with disease progression. Interestingly, PD-1 expression was constantly retained on CXCR5^+^CD8^+^ T cells while significantly decreased on CXCR5^−^CD8^+^ T cells after successful antiretroviral treatment in chronic HIV-infected patients. PD-1^+^CXCR5^+^CD8^+^ T cells may represent a novel therapeutic strategy for the disease control. Kleinman et al. provide an overview of the phenotype, development/homeostasis and function of Treg and follicular regulatory (Tfr) CD4 T cells as well as strategies for their *in vivo* manipulation in an effort to eliminate the virus. The frequency of Treg cells is increased in HIV infection while there are susceptible to viral infection. Therefore, Tregs cells can affect HIV pathogenesis by (i) suppressing antiviral immune responses and (ii) contributing to viral reservoir formation. Huot et al. discuss the differences that exist between non-pathogenic SIV infection in natural hosts and pathogenic HIV/SIV infection in humans and macaques regarding virus target cells and replication dynamics in LNs. They emphasized on the NK cell-mediated control and the impact that these insights on viral dynamics and host responses in LNs of natural hosts have for the development of strategies toward HIV cure. Estes et al. provide insights of imaging techniques which help to characterize the compartmentalization of highly specialized immune and stromal lymph node cell populations, the local interplay between the virus and host cells with respect to viral persistence, immune responses, tissue structure and pathologies, and changes to the surrounding milieu and function of immune cells. Merging imaging platforms with other cutting-edge technologies could lead to novel findings regarding the phenotype, function, and molecular signatures of particular immune cell targets, further promoting the development of new antiviral treatments and vaccination strategies.

A major obstacle in the fight against HIV is the establishment of a viral reservoir compartment that persists even under long-term successful antiretroviral treatment. Bronnimann et al. provide a comprehensive review of biological factors contributing to the role of B cell follicle as a major site for actively transcribed virus. The role of particular cell types, including Tfh, follicular CD8, Follicular Dendritic Cells (FDCs), γδT, and NK cells in this process is discussed. An overview of “cure strategies” targeting the virus within the follicular area are also presented. Several reports have revealed the importance of Tfh cell compartment for the viral propagation and reservoir establishment. Dave et al. discuss the unique contribution of cervical lymph nodes (CLNs) in establishing/maintaining viral reservoir. CLNs is a draining site for meningeal and nasal lymphatics and virus could access these anatomical sites through CNS and conventional DCs or CD4 T cells infected within the CNS compartment. Similar to peripheral LNs, CLN FDC network can contribute to viral spreading and reservoir establishment. The authors also discuss possible ways to purge the virus from these anatomical sites. Novel, more sophisticated strategies are needed for the elimination of the virus, especially form sites like follicular areas. Chimeric Antigen Receptor genetically engineered T cells (CAR) represents a promising immunotherapy for hematological tumors and actively pursued for solid tumors too. Aid et al. discuss specific cellular signaling molecules and transcriptional factors as regulators of lymph node Tfh cell development. System biology tools can provide valuable information to this regard. The authors describe molecular pathways and particular molecules that could promote the infection of Tfh cells and establishment of the viral reservoir. Further investigation of Tfh cell biology could lead to novel strategies for the efficient depletion of HIV from this T cell compartment. Besides Tfh cells, the role of FDCs in capturing virions and contribute to viral spreading is well-established. Haran et al. provide original data describing the construction of anti-SIV CAR/CXCR5 T cells. The engineered cells have the capacity to populate the follicular area in an *ex vivo* B cell follicle migration assay while retaining their viral suppression activity. The provided data further support the development of CAR technology as an alternative approach for virus elimination in the follicular areas. Wang and Xu review the epigenetic regulation of GC responses, especially for GC B and Tfh cell under normal and during chronic HIV/SIV infection. Ellegård et al. provide original data showing that dendritic cells, natural killer cells, and T cells play critical roles during primary HIV-1 exposure at the mucosa, where the viral particles become coated with complement fragments and mucosa-associated antibodies. The microenvironment together with subsequent interactions between these cells and HIV at the mucosal site of infection will determine the quality of immune response that ensues adaptive activation. George and Mattapallil reviewed the role of IFN-alpha subtypes in HIV infection and discuss the possibility that certain subtypes could be potential adjuncts to a “shock and Kill” or therapeutic vaccination strategy that can lead to better control of the latent reservoir and subsequent functional cure while Wang et al. discuss the potential role of IFN-I as regulator of innate and adaptive immunity, including Tfh cells, in chronic HIV infection and the therapeutic strategies targeting IFN-I in infected individuals.

This eBook provides a comprehensive presentation of recent published work on lymph node and especially Tfh cell dynamics in HIV infection and we hope that it will be useful for our further understanding of how such dynamics affect the interplay between virus and host as well as for the discovery of novel therapeutic targets in the fight against HIV.

## Author Contributions

All authors listed have made a substantial, direct and intellectual contribution to the work, and approved it for publication.

### Conflict of Interest Statement

The authors declare that the research was conducted in the absence of any commercial or financial relationships that could be construed as a potential conflict of interest.
